# Human neuronal uncoupling proteins 4 and 5 (UCP4 and UCP5): structural properties, regulation, and physiological role in protection against oxidative stress and mitochondrial dysfunction

**DOI:** 10.1002/brb3.55

**Published:** 2012-07

**Authors:** David B Ramsden, Philip W-L Ho, Jessica W-M Ho, Hui-Fang Liu, Danny H-F So, Ho-Man Tse, Koon-Ho Chan, Shu-Leong Ho

**Affiliations:** 1 School of Medicine and School of Biosciences, University of BirminghamUnited Kingdom; 2 Division of Neurology, Department of Medicine, University of Hong KongHong Kong, PR China; 3 Research Centre of Heart, Brain, Hormone and Healthy Aging (HBHA), University of Hong KongHong Kong, PR China

**Keywords:** Energy homeostasis, mitochondrial dysfunction, neurodegeneration, neuroprotection, oxidative stress, uncoupling proteins

## Abstract

Uncoupling proteins (UCPs) belong to a large family of mitochondrial solute carriers 25 (SLC25s) localized at the inner mitochondrial membrane. UCPs transport protons directly from the intermembrane space to the matrix. Of five structural homologues (UCP1 to 5), UCP4 and 5 are principally expressed in the central nervous system (CNS). Neurons derived their energy in the form of ATP that is generated through oxidative phosphorylation carried out by five multiprotein complexes (Complexes I–V) embedded in the inner mitochondrial membrane. In oxidative phosphorylation, the flow of electrons generated by the oxidation of substrates through the electron transport chain to molecular oxygen at Complex IV leads to the transport of protons from the matrix to the intermembrane space by Complex I, III, and IV. This movement of protons to the intermembrane space generates a proton gradient (mitochondrial membrane potential; MMP) across the inner membrane. Complex V (ATP synthase) uses this MMP to drive the conversion of ADP to ATP. Some electrons escape to oxygen-forming harmful reactive oxygen species (ROS). Proton leakage back to the matrix which bypasses Complex V resulting in a major reduction in ROS formation while having a minimal effect on MMP and hence, ATP synthesis; a process termed “mild uncoupling.” UCPs act to promote this proton leakage as means to prevent excessive build up of MMP and ROS formation. In this review, we discuss the structure and function of mitochondrial UCPs 4 and 5 and factors influencing their expression. Hypotheses concerning the evolution of the two proteins are examined. The protective mechanisms of the two proteins against neurotoxins and their possible role in regulating intracellular calcium movement, particularly with regard to the pathogenesis of Parkinson's disease are discussed.

## Family Membership

Members of the mitochondrial solute carrier 25 (SLC25) family of proteins function as transporters of a large variety of molecules, including ATP, ADP, amino acids (e.g., glutamate, aspartate, lysine, histidine, arginine, ornithine, and citrulline), malate, and calcium (Palmieri 1994; Bassi et al. 2005). All of the SLC25s (in total over 40 members) are localized at the inner mitochondrial membrane with the exception of SLC25A17 (Palmieri 2004) and 14 of the SLC25 members are located in the central nervous system (CNS). Within this SLC25 family exists a subfamily of five proteins (so-called uncoupling proteins [UCPs]), which transport protons directly from the intermembrane space to the matrix—SLC25A7 (UCP1), SLC25A48 (UCP2), SLC25A9 (UCP3), SLC25A27 (UCP4), and SLC25A14 (UCP5) (Kim-Han and Dugan 2005). This short review concentrates on two UCPs that are expressed principally in the CNS: UCP4 and UCP5.

## General Properties of UCPs

Much of our understanding of the structure and functions of UCPs stems from work on UCP1 to 3. UCP4 and 5 have the characteristic structure of the other UCPs. Their single amino acid chain can be viewed as configured into three cassettes, each with two membrane-spanning alpha helices. Both the amino and carboxy terminals are positioned in the intermembrane space, but the mechanism of transport of protons is still not fully resolved (Jastroch et al. 2010). In addition to transporting protons, these UCPs have a purine nucleotide binding site located projecting into the intermembrane space. The purine nucleotides ATP, ADP, GTP, and GDP are inhibitors of uncoupling activity (Klingenberg 1988; Huang and Klingenberg 1995; Xia et al. 2008), whereas superoxide is an activator (Echtay et al. 2002).

## The Importance of Uncoupling

**Figure 1:**
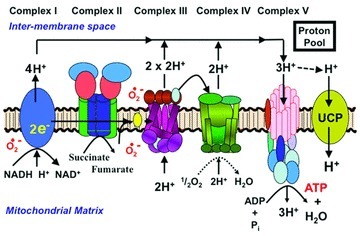
Oxidative phosphorylation in mitochondrial electron transport chain (ETC), and proton leak via uncoupling proteins (UCPs). UCP dissipates mitochondrial membrane potential by facilitating proton leak across the inner membrane, thereby minimizing superoxide (O_2_^•−^) formation from undesirable interaction between molecular oxygen (O_2_) and high-energy electrons (e^−^).

Neurons derive their energy in the form of ATP from the oxidation of glucose. Initially glucose is oxidized to pyruvate in the cytosol through glycolysis. The pyruvate is transported into the mitochondrial matrix where it is converted to acetyl co-enzyme A (ACoA) by pyruvate dehydrogenase. AcoA enters the citric acid cycle, in which the acetyl group is oxidized to carbon dioxide. In the citric acid cycle, NAD^+^ is reduced to NADH and FAD is reduced to FADH_2_. NADH and FADH_2_ are the substrates for oxidative phosphorylation (Fig. 1). Oxidative phosphorylation is carried out by five multiprotein complexes. Complexes I–IV form an electron transport chain (ETC) where electrons are donated to oxygen at Complex IV. Protons are pumped from the matrix to the intermembrane space by Complex I, III, and IV. This movement of protons to the intermembrane space generates an electrochemical gradient or proton motive force that is used by Complex V to drive the conversion of ADP to ATP. Three protons passing back from the intermembrane space to the matrix are necessary to convert one molecule of ADP to ATP (Mitchell 1961, 1966). Not all electrons entering the ETC are passed to Complex IV. Some electrons escape to oxygen at Complexes I and III, giving rise to the formation of harmful reactive oxygen species (ROS) (Skulachev 1996, 1997, 1998; Korshunov et al. 1997). ROS formation is particularly high when Complex I is inhibited (Votyakova and Reynolds 2001). Although cells have evolved ways of dealing with ROS once formed a means of preventing or minimizing ROS formation is energetically advantageous. Thus, a leak of protons back to the matrix, bypassing Complex V, results in a major reduction in ROS formation while having a minimal effect on ATP synthesis. A slight decrease in the potential difference across inner mitochondrial membrane has been shown to inhibit H_2_O_2_ formation by 70% (Hansford et al. 1997; Votyakova and Reynolds 2001; Echtay 2007) (Fig. 1).

Therefore, it is not surprising that primitive life forms evolved a protein that could bring about such a regulated leak, thereby uncoupling the electron transport and proton export aspects of the oxidative phosphorylation process from ATP synthesis. However, the ability of a protein to undertake uncoupling does not rule out the possibility that other tasks could be performed also, such as regulation of intracellular calcium or synaptic function (Jezek 2002; Andrews et al. 2005).

### UCPs 4 and 5

Human UCP4 was first identified as a novel member of the human UCP family (Mao et al. 1999). The gene encoding the protein is located on chromosome 6p11.2-q12. It gives rise to a single transcript, which is translated into a protein of 323 amino acids—approximately 34 kDa (Mao et al. 1999). The gene encoding UCP5 is on Xq24. UCP5 was first described and named as brain mitochondrial carrier protein-1 (BMCP1) (Sanchis et al. 1998). Three isoforms of human UCP5 have been identified; long form containing 325 amino acids (UCP5L), short form containing 322 amino acids with the deletion of Val-Ser-Gly (VSG) at position 23–25 (UCP5S), and short insert form containing 353 amino acids with VSG deleted but insertion of 31 amino acids between transmembrane domains III and IV (UCP5SI) (Kondou et al. 2000; Yu et al. 2000b; Kim-Han et al. 2001; Lengacher et al. 2004; Palmieri 2004; Echtay 2007). Although UCPs 4 and 5 are principally expressed in the CNS, they are expressed in other tissues to some extent, for example, UCP5 short form with insert is found in human skeletal muscle (Yang et al. 2002).

In an attempt to define the structural characteristics that are unique to UCPs, the primary structures of 19 mitochondrial proteins were compared in 10 plant and animal species, which have proton-pumping capability (Jezek and Urbankova 2000). Common amino acid sequences were identified in the first, second, and fourth transmembrane helices, the matrix segment between the second and third helices, and the purine nucleotide binding site that possess high homology. These sequences they termed “UCP signatures.” Further analysis of these UCP signatures led a proposal describing the evolution of the five human UCPs from a common ancestral gene (Hanak and Jezek 2001). They proposed that:

1. the ancestral gene (possibly encoding a primitive ADP/ATP transporter) gave rise to two branches, from the first of which UCP4 evolved, whereas the other four UCPs evolved from a second branch,
2. UCP4 is the most closely related to this ancestral gene,
3. UCP5 originated from an early division of the second branch,
4. UCP 1, 2, and 3 appeared later in evolution, are closely related, and derived from a separate division of the second branch compared with the one which gave rise to UCP5.

This hypothesis was rebutted by Sokolova and Sokolov (2005) who proposed that UCPs diverged from an ancestral gene into at least three genetically distinct forms very early in the evolution (Sokolova and Sokolov 2005). The three forms correspond to the clades identified by the phylogenetic analysis. Clade 1 contains vertebrate UCPs 1, 2, and 3. Clade 2 contains vertebrate UCP5 and a UCP5 homologue from *Drosophila melanogaster*. Clade 3 includes UCP4 from mammals and UCP4a and UCP4b from *D. melanogaster*. They identified and proposed that an invertebrate UCP6 is closest to the ancestral gene that also gave rise to vertebrate UCP1, 2, and 3. Both the above hypotheses may well be modified as more complete genomes are elucidated. Nevertheless, both hypotheses illustrate the distinctly different characters of UCP 4 and 5 compared with UCP1 to 3. Both the difference of UCP4 and 5 from the other UCPs, and further understanding of the process of UCP evolution was illustrated by the observation that some avian species lacked UCP1 and 2 (Emre et al. 2007). The dissimilarity of UCP4 from the other UCPs was further demonstrated by the properties of pure preparations of the five human UCPs (Ivanova et al. 2010). When they were reconstituted in detergents and in stable small unilamellar vesicles, all the UCPs formed dominantly helical conformations in negatively charged phospholipid vesicles, but UCP4 had a different helical profile that may be related to its less associated form. In addition, the binding of purine nucleotides to UCP4 was different to that exhibited by the other UCPs.

### Expression of UCP4 and 5 in the CNS

**Figure 2:**
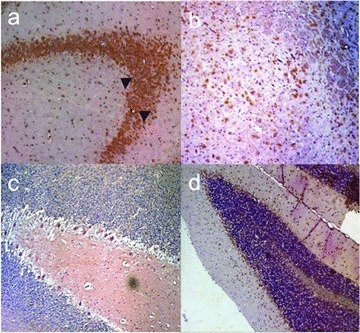
Expression of UCP4 in brain, (a) rat hippocampus; (b) rat substantia nigra; (c) human cerebellum*; (d) rat cerebellum (solid arrowhead: brown denotes positive staining). *Human postmortem brain sections were obtained from the Parkinson's Disease Society Brain Bank of UK. Research ethics on immunohistochemistry of human brain samples was approved by the Institutional Review Board of the University of Hong Kong/Hospital Authority Hong Kong West Cluster (HKU/HA HKW IRB) (IRB ref. number UW 06–108 T/1133), complying with the Declaration of Helsinki and acts in accordance to local regulations by Hong Kong Hospital Authority and the University policies.

As mentioned above, both UCP4 and 5 are expressed primarily in the brain. Although there is no detailed description of the regional distribution in the human brain, if one assumes that this follows the pattern in rodents, there are likely to be marked differences in the level of expression in different brain areas. In mouse, UCP5 is strongly expressed in amygdala, dorsomedial hypothalamic nucleus, hippocampus, paraventricular thalamic nucleus, mediodorsal thalamic nucleus, and ventromedial hypothalamus (Sanchis et al. 1998; Huang et al. 2011). UCP4 appears in neurons and to a lesser extent in astrocytes of murine neuronal tissue as early as days 12–14 of embryonic development (Smorodchenko et al. 2009). UCP4 mRNA was found to be expressed in inner ear ganglia (Kitahara et al. 2004), and neurosensory cells such as hair cells of the inner ear and mechanosensitive Merkel cells in skin express a significant amount of UCP4 (Smorodchenko et al. 2011). Our own results for UCP4 in rat brain showed that it was strongly expressed in pyramidal cells in the hippocampus (Fig. 2a) and cortex, and in a wide range of cells in substantia nigra (Fig. 2b) and striatum, and Purkinje cells in cerebellum (Fig. 2d). In the only human brain tissue we studied, UCP4 was found to be expressed in Purkinje cells (Fig. 2c), in line with the findings in rat brain. Nevertheless, significant differences in levels of expression of UCP4 and 5 in brain between rats and mice may make any cross-species assumptions (e.g., from rat or mouse to human) based solely on analogy to be misleading. UCP5 expression dominates in rat brain in contrast to 10-fold higher UCP4 expression in mouse brain (Alan et al. 2009). Thus, a detailed investigation of expression of UCP4 and 5 and their mRNA in human CNS would be timely.

### Protective effects of UCP4 and 5

The marked differences between the amino acid sequences of UCP4 and 5 compared with those of UCP1 to 3, and the limited tissue distribution of UCP4 and 5 suggest that they may have different roles compared with UCP1 to 3. Nevertheless, whatever specialist roles these two proteins may possess, both UCP4 and 5 pass protons through the inner mitochondrial membrane to the matrix. Thus, both UCP4 and 5 perform the essential function of an uncoupler of oxidative phosphorylation. This process is accompanied by a reduction in oxidative stress, and consequentially both exert a protective influence on cells exposed to mitochondrial toxic insults (Zhang et al. 2006). We have shown that SH-SY5Y cells (a human catecholaminergic neuronal cell line) that overexpress either UCP4 or UCP5 are more resistant, in terms of survival and levels of ROS, to the effects of 1-methyl-4-phenylpyridinium ion (MPP^+^, a selective dopaminergic toxin) (Ho et al. 2006), dopamine (Chu et al. 2009; Kwok et al. 2010), and hydrogen peroxide than similarly treated control cells with endogenous levels of UCP expression. In addition, the protective action of UCP4 has been shown against Complex II specific toxin, 3-nitropropionic acid (Wei et al. 2009). These actions were proposed to be a consequence of a reduction in ROS levels, which is in accord with the concept of mild uncoupling being a protective mechanism. Given the relatively low levels of endogenous expression of UCP4 and 5 even in neurons where they are expressed, this uncoupling action is unlikely to generate large amounts of heat (Yu et al. 2000a). However, it has been suggested, as in the case of UCP2, that whatever heat is generated by UCPs may slightly increase the speed of synaptic transmission (Horvath et al. 1999). Tables 1 and 2 summarize some functional properties of UCP4 and UCP5.

**Table 1:** Summary of evidence demonstrating UCP4 function

Functions of UCP4	Citations
**Energy homeostasis**	
UCP4 increases mitochondrial Complex II activity in SH-SY5Y neuroblastoma cells.	Ho et al. 2012
UCP4 regulates mitochondrial Complex II activity via succinate import in *Caenorhabditis elegan*.	Pfeiffer et al. 2011
UCP4 overexpression improves fatty acid oxidation and insulin sensitivity in L6 myocytes.	Gao et al. 2011
UCP4 increases glucose uptake and shifting the mode of ATP production from mitochondrial respiration to glycolysis in PC12 cells.	Liu et al. 2006
Ectopic expression of UCP4 reduces mitochondrial membrane potential in MCF-7 cells.	Mao et al. 1999
**Gene regulation**	
NF-κB p50/c-Rel signal pathway induces UCP4 expression in SH-SY5Y cells.	Ho et al. 2010
UCP4 expression is decreased in Parkinsonian DJ-1 knockout mice.	Guzman et al. 2010
UCP4 transcription is genomically regulated by T3 and cAMP.	Dorsa et al. 2010
Methionine restriction increases UCP4 expression in Wistar rats.	Naudi et al. 2007
UCP4 gene transcription is induced by mitochondrial toxin, MPP^+^, in SK-N-SH cells.	Ho et al. 2005
Ketogenic diet increases UCP4 expression in SD rats.	Sullivan et al. 2004
**Neuroprotection**	
UCP4 preserves mitochondrial depolarization and decreases oxidative stress against MPP^+^ toxicity in SH-SY5Y cells, and potential functional compensation with UCP2.	Chu et al. 2009
UCP4 mediates Complex II specific bioenergetics adaptation and cell survival against 3-NP toxicity via activation of ERK in PC12 cells.	Wei et al. 2009
UCP4 regulates calcium homeostasis and apoptosis in PC12 cells.	Chan et al. 2006
Brain UCP4 transcript was increased after acute cold exposure in FVB-N mice implicating regulation.	Yu et al. 2000b
**Relationship with disease pathologies**	
Association of a UCP4 (SLC25A27) haplotype with ultraresistant schizophrenia.	Mouaffak et al. 2011
A homozygous genetic variant of mitochondrial UCP4 affects the occurrence of leukoaraiosis.	Szolnoki et al. 2010
A homozygous genetic variant of mitochondrial UCP4 exerts protection against the occurrence of multiple sclerosis.	Szolnoki et al. 2009
Association of a human UCP4 SNP with schizophrenia.	Yasuno et al. 2007

**Table 2:** Summary of evidence demonstrating UCP5 function

Functions of UCP5	Citations
**Energy homeostasis**	
Loss of UCP5 modifies the energy balance and increases free radicals through upregulation of UCP3.	Senapedis et al. 2011
Knockdown of UCP5 potentiates mitochondrial depolarization, ATP deficiency, and induces apoptosis.	Ho et al. 2006
UCP5 knockout flies are highly sensitive to starvation stress to maintain metabolic homeostasis.	Sanchez-Blanco et al. 2006
**Gene regulation**	
UCP5 expression is decreased in Parkinsonian DJ-1 knockout mice.	Guzman et al. 2010
UCP5 transcription may be genomically regulated by T3 and cAMP.	Dorsa et al. 2010
Hydrogen peroxide increases expression of UCP 5 in colon cancer cells.	Santandreu et al. 2009
Saturated fatty acids increases but insulin suppresses UCP5 expression in bovine mammary epithelial cells.	Yonezawa et al. 2009
UCP5 gene transcription is induced by mitochondrial toxin, MPP^+^, in SK-N-SH cells.	Ho et al. 2005
Ketogenic diet increases UCP5 expression in SD rats.	Sullivan et al. 2004
Brain UCP5 transcript was increased after acute cold exposure in FVB-N mice implicating thermoregulation.	Yu et al. 2000b
**Neuroprotection**	
UCP5 preserves mitochondrial membrane potential, ATP levels, and reduces oxidative stress against MPP^+^ and dopamine toxicity in SH-SY5Y cells.	Kwok et al. 2010
**Relationship with disease pathologies**	
Associations between genetic variants in UCP5 SNP and atherosclerotic plaque.	Dong et al. 2011
UCP5 was significantly elevated in the ischemic lesions of stroke patient brain.	Nakase et al. 2007

### Some factors that affect expression

In nonneuronal tissues, fatty acids upregulate both UCP activity and expression. Saturated fatty acids have been shown to upregulate UCP5 expression in bovine mammary epithelial cells (Yonezawa et al. 2009). Although a high-fat diet has also been shown to increase expression of UCP5 mRNA by a factor of 1.8 in mouse liver, it had no effect on the levels of UCP4 and UCP5 mRNAs in brain (Yu et al. 2000b). The same authors showed that within the brain, the mRNA levels of UCP4 and 5 were modulated by environmental temperature. A low environmental temperature (4°C) induced a rise in both UCP4 and UCP5 transcripts. Whether these rises indicate a thermoregulatory role for the proteins is uncertain. The phenomena may be a nonspecific stress effect. Other factors such as ROS (Santandreu et al. 2009), caloric restriction (Liu et al. 2006), exposure to toxins (Ho et al. 2005), a ketogenic diet (Sullivan et al. 2004), and methionine-restricted diet (Naudi et al. 2007) also upregulate expression of either or both the proteins, whereas insulin downregulates expression of UCP4 and 5 (Yonezawa et al. 2009) and GDP inhibits activity of UCP4 (Liu et al. 2006). Surprisingly, in view of the effects of UCP4 on glucose metabolism, hypoxia had no effect on expression of the protein (Smorodchenko et al. 2011), although UCP5 is reported to be elevated in areas of human brain with ischemic lesions (Nakase et al. 2007). Estrogen treatment of an estrogen receptor positive breast cancer cell-line (MCF-7) downregulated both UCP4 and 5 (Sastre-Serra et al. 2010). In contrast, old female rats had higher UCP4 and 5 levels in mitochondria compared with similarly aged male animals, which may explain in part the lower oxidative stress in female brains (Guevara et al. 2009, 2011). Interestingly, in a human study of over 100 metastatic breast cancers, UCP4 expression was positively correlated with estrogen receptor and progesterone receptor expression (*P* < 0.0001 for both), with lymph node metastases (*P*= 0.005), as well as positivity for p53 (*P* < 0.0001) and Ki-67 (*P* < 0.0001). UCP4 expression was correlated negatively with Bcl-2 expression (*P*= 0.001). Furthermore, UCP4 expression was correlated with aneuploid tumors (*P*= 0.002) (Gonidi et al. 2011).

### Mechanisms of regulation

We have listed above some factors that can affect transcription of UCP4 and 5 genes, but there is no detailed description of how expression of either protein is regulated. To address this issue, we carried out a brief in silico analysis of the first 3000 bases extending upstream of the transcription initiation sites in human using the MatInspector program. Although such an analysis only identifies potential *cis*-acting factors, it appears that the two genes are likely to be regulated in very different ways. Table 3 lists some potential sites particularly connected with their expression in the CNS. In the 5′-flanking region of the UCP4 sites for Lim homeobox domain, BmPOU factors, and other homeobox transcription factors are abundantly evident. In contrast, cAMP response element binding protein (CREBP) is more common in the 5′-flanking region of UCP5 gene sites, although both regions contained significant numbers. In a detailed in silico investigation, Dorsa et al. (2010) found that both UCP and UCP5 were likely to be strongly regulated by 3,5,3′-triiodothyronine and by CREBP signaling. In accord with this, activation of extracellular signal regulated kinases (ERKs) was necessary and sufficient to mediate the effects of UCP4 on glucose utilization in PC12 cells exposed to 3-nitropropionic acid, a Complex II specific inhibitor (Wei et al. 2009). Pharmacological inhibition of ERKs reduced the activation of CREBP and the authors hypothesized that CREBP signaling contributes to UCP4-dependent cell death rescue.

**Table 3:** In silico analysis for potential transcription factor binding sites in 5′-flanking regions of human UCP4 and UCP5 genes

Potential sites for factors	Number in UCP4	Number in UCP5	References describing some of the properties of these factors
AP1	4	0	Yoneda et al. 2001
Bicoid-like homeodomain	12	6	Quentien et al. 2011
BmPOU	24	12	Mathis et al. 1992
CAMP-responsive protein	8	15	Sakamoto et al. 2011
EGR/nerve growth factor	4	10	O’Donovan et al. 1999
Homeobox transcription factors	22	5	Cheng and White 2011
MYT1 C2HC zinc finger protein	11	4	Cheng and White 2011

Another site that appears to be important in the modulation of UCP4 gene transcription is a NF-κB site in the first 1000 bases adjacent to the transcription initiation site. We have shown that site-directed mutation of this site severely reduced the ability to stimulate gene transcription in response to NF-κB and thus ameliorate the response to oxidative stress caused by MPP^+^ (Ho et al. 2010). In a subsequent study, we have shown that this particular NF-κB dimer binding to this site is composed of c-Rel and p50 monomers (J. W. M. Ho, P. W. L. Ho, and S. L. Ho, unpubl. data). NF-κB dimers may be composed of any of p50, p52, p65, RelB, and c-Rel monomer. p65-containing dimers are associated with the stimulation of apoptotic cell death (Pizzi et al. 2002; Lanzillotta et al. 2010), whereas c-Rel-containing dimers are associated with cell survival pathways (Pizzi et al. 2005; Pizzi and Spano 2006).

The complexity of interrelationship between modulation of energy supply by UCPs on intracellular functioning is beginning to be elucidated. Knockdown of UCP5 was found to affect energy balance and led to increased ROS and upregulation of UCP3, then via increased c-Jun N-terminal kinase 1 (JNK1) kinase activity and Akt dephosphorylation to modulation of FOXO localization (Senapedis et al. 2011). Thus, modulation of the expression of one UCP5 can affect expression of another UCPs and has further consequences for cell signaling and function.

### Enigmatic UCP4

UCP5 acts like a typical UCP. Knockdown of UCP5 reduced the ability of cells to withstand the toxic actions of MPP^+^ (Ho et al. 2006), and overexpression of UCP5 resulted in reduced mitochondrial membrane potential (MMP), reduced intracellular ATP content, and reduced levels of ROS (Sanchis et al. 1998; Kwok et al. 2010). As a consequence, all our SH-SY5Y clones that overexpress the protein replicate more slowly than the untransfected control cells. Some reports on UCP4 described similar effects of overexpression on MMP, ATP content, and ROS levels (Yu et al. 2000b; Liu et al. 2006). Therefore, it was unsurprising that after overexpressing UCP4 in SH-SY5Y cells, we found MMP and intracellular ATP were increased and the rate of replication was faster than in the control cells (Chu et al. 2009). Subsequently, we found that knockdown of UCP4 expression in SH-SY5Y cells by siRNA transfection lowered MMP and increased ROS levels (unpubl. data). Contrary to the findings of Liu et al. (2006) and Wei et al. (2009), we found overexpression of UCP4 did not shift glucose metabolism toward glycolysis and away from oxidative phosphorylation (Chu et al. 2009). Our findings are completely at variance to what one would expect of a classical UCP and were met with disbelief by some initial reviewers. Such a divergence of findings needs an explanation. First, we would point out that UCP4 is distinctly different from the other UCPs in that it evolved at a very early stage and along a different path than the other UCPs. The difference is also evident in structural characteristics, and binding properties as mentioned earlier. Nevertheless, this would not account for the divergence of results of the different groups. There are methodological differences that may be relevant. We overexpressed the human protein in a human cell line of catecholaminergic origin, whereas other groups have used either nonhuman neuronal cells or human nonneuronal cells. Marked differences in expression UCP4 and UCP5 occur in different but quite closely related species (Alan et al. 2009). The cell-specific nature of the consequences of UCP4 overexpression was previously illustrated (Gao et al. 2010, 2011). These authors found that overexpression of UCP4 in 3T3-adipocytes impaired insulin sensitivity (Gao et al. 2010), whereas in L6-myocytes UCP4 overexpression improved it (Gao et al. 2011). In addition, it is difficult to compare levels of overexpression of the protein in the clones of different groups of investigators. It has been suggested that our high levels of UCP4 expression may result in mitochondria being packed with misfolded protein. In view of the fact that our UCP4 overexpressing SH-SY5Y cells have faster replication rates, higher ATP content, and lower ROS levels (Chu et al. 2009), the presence of substantial quantities of a misfolded protein seems unlikely. Furthermore, knockdown of endogenous UCP4 in SH-SY5Y cells also yields unanticipated results in that MMP is significantly lower (J. W. M. Ho, P. W. L. Ho, and S. L. Ho, unpubl. data). Subsequent studies showed that overexpression of UCP4 resulted in increased Complex II activity (Ho et al. 2012). The mechanism of this stimulation is associated with protein–protein interaction between UCP4 and Complex II (Ho et al. 2012), which mirrors similar interaction reported by Pfeiffer et al. (2011) in *Caenorhabditis elegans*, where they showed that UCP4 controls Complex II mediated oxidative phosphorylation through succinate transport (Pfeiffer et al. 2011). Knockdown of UCP4 reduced the contribution of Complex II to ATP synthesis by reducing succinate availability.

### UCPs 4 and 5 and disease

The ability of UCPs to modulate ROS formation has prompted searches for connections with both neurological and nonneurological disease states, for example, SNPs in the UCP5 gene are associated with atherosclerotic plaque formation (Dong et al. 2011). SNPs in and around the UCP2 and UCP4 genes were investigated in subjects with schizophrenia. A significantly increased risk (7.6-fold) of developing the disease was found in homozygous individuals possessing risk alleles at rs660339 and rs10807344, which points to the involvement of these two UCPs in the etiology of schizophrenia (Mouaffak et al. 2011). This association between UCP2 and UCP4 with the etiology of schizophrenia is in accord with the results of an earlier study by Yasuno et al. (2007). The expression of UCP4 has been found to be increased in cell culture and a murine model of ALS, where neuronal death is attributed to oxidative stress. In contrast, in a Drosophila model of Huntington's disease, UCPs were shown protect glia rather than neurons (Besson et al. 2010).

### The relevance of UCPs to Parkinson's disease

Our interest in UCPs is based on Parkinson's disease (PD). Although much research into the etiology of PD has taken place, this has very largely focused on the role of mutant proteins in familial PD. Fourteen or more genetic loci have been identified and the pathophysiological action of mutant proteins encoded by genes in these loci are being elucidated. Nevertheless, the contribution of genetic mutation to the overall burden of PD is very small. The most optimistic estimates that are around 5–10% of all PD cases are due to such mutations. Of the remaining 90–95%, arguments have been made to show that environmental factors such as pesticide exposure are involved. This may be the case in a certain number of instances. Nonetheless, whether a combination of a genetic susceptibility and toxin exposure accounts for the majority of cases of sporadic PD remains unclear. If these two factors are not the cause, it would be logical to look at the physiology of the brain itself. The hypothesis put forward by Surmeier and colleagues uses this as a basis (Guzman et al. 2009, 2010). They have pointed out that dopaminergic neurons of the substantia nigra have a relatively rare mechanism of autonomous pacemaking. During pacemaking in these neurons, calcium entry occurs via L-type Ca^2+^ channels with a Ca_v_1.3 pore-forming subunit. They proposed that the relatively open nature of these channels allows greater calcium entry, which in turn incurs a high metabolic cost in terms of ATP to maintain tight control of intracellular calcium by the endoplasmic reticulum and the mitochondria. The high ATP requirement, which has to be met by mitochondrial synthesis, results in greater ROS production that ultimately overwhelms neuronal antioxidant defenses and leads to cell death. Whether the complete scenario envisaged by Surmeier et al. (2011) is correct awaits further substantiation. Nonetheless, there is long-established evidence for the role of calcium in the control of mitochondrial substrate oxidation (Hansford and Zorov 1998) and in neuronal cell death (Stout et al. 1998). This evidence has led to the possible involvement of UCPs, and UCP4 in particular. Chan et al. (2006), using PC-12 cell clones which either express UCP4 or not, have shown that UCP4 is a potent influence on store-operated calcium entry and on mitochondrial sequestration of calcium (Chan et al. 2006). They proposed that prevention of calcium overload by UCP4 inhibition of store-operated calcium channels, with consequent reduction in oxidative stress, reduces the likelihood of calcium-primed cell death. Furthermore, in a DJ-1 knockout mouse model, the expression of both UCP4 and UCP5 was downregulated and DJ-1 modulated the magnitude of the response of these two UCPs to oxidative stress (Guzman et al. 2010). Although the route of calcium entry in the Surmeier hypothesis (via L-type Ca^2+^ channels) and calcium entry via store-operated channels are entirely different, the hypotheses proposed in both studies center on the ability of the mitochondrion to regulate calcium levels, where superoxide actions on UCP4 and UCP5 expression play a key role (Wu et al. 2009). Given that the expression of the two proteins is likely to be normally distributed, it would be interesting to determine levels of expression of the proteins in relation to the development of PD. A further extension of this would be to determine whether xenobiotic agents are able to modulate expression either detrimentally or deliberately beneficially.

In conclusion, although neuronal UCP4 and 5 are relatively unknown and unexplored entities in PD, the properties of the two, particularly of UCP4, make them interesting potential players in the etiology of the disease and also possible targets for drug intervention therapy.

## References

[b1] Alan L, Smolkova K, Kronusova E, Santorova J, Jezek P (2009). Absolute levels of transcripts for mitochondrial uncoupling proteins UCP2, UCP3, UCP4, and UCP5 show different patterns in rat and mice tissues. J. Bioenerg. Biomembr.

[b2] Andrews ZB, Diano S, Horvath TL (2005). Mitochondrial uncoupling proteins in the CNS: in support of function and survival. Nat. Rev. Neurosci.

[b3] Bassi MT, Manzoni M, Bresciani R, Pizzo MT, Della Monica A, Barlati S, Monti E, Borsani G (2005). Cellular expression and alternative splicing of SLC25A23, a member of the mitochondrial Ca2+-dependent solute carrier gene family. Gene.

[b4] Besson MT, Dupont P, Fridell YW, Lievens JC (2010). Increased energy metabolism rescues glia-induced pathology in a Drosophila model of Huntington's disease. Hum. Mol. Genet.

[b5] Chan SL, Liu D, Kyriazis GA, Bagsiyao P, Ouyang X, Mattson MP (2006). Mitochondrial uncoupling protein-4 regulates calcium homeostasis and sensitivity to store depletion-induced apoptosis in neural cells. J. Biol. Chem.

[b6] Cheng Z, White MF (2011). Targeting Forkhead box O1 from the concept to metabolic diseases: lessons from mouse models. Antioxid. Redox Signal.

[b7] Chu AC, Ho PW, Kwok KH, Ho JW, Chan KH, Liu HF, Kung MH, Ramsden DB, Ho SL (2009). Mitochondrial UCP4 attenuates MPP+- and dopamine-induced oxidative stress, mitochondrial depolarization, and ATP deficiency in neurons and is interlinked with UCP2 expression. Free Radic. Biol. Med.

[b8] Dong C, Della-Morte D, Wang L, Cabral D, Beecham A, Mcclendon MS, Luca CC, Blanton SH, Sacco RL, Rundek T (2011). Association of the sirtuin and mitochondrial uncoupling protein genes with carotid plaque. PLoS One.

[b9] Dorsa KK, Santos MV, Silva MR (2010). Enhancing T3 and cAMP responsive gene participation in the thermogenic regulation of fuel oxidation pathways. Arq. Bras. Endocrinol. Metabol.

[b10] Echtay KS (2007). Mitochondrial uncoupling proteins–what is their physiological role?. Free Radic. Biol. Med.

[b11] Echtay KS, Roussel D, St-Pierre J, Jekabsons MB, Cadenas S, Stuart JA, Harper JA, Roebuck SJ, Morrison A, Pickering S, et al (2002). Superoxide activates mitochondrial uncoupling proteins. Nature.

[b12] Emre Y, Hurtaud C, Ricquier D, Bouillaud F, Hughes J, Criscuolo F (2007). Avian UCP: the killjoy in the evolution of the mitochondrial uncoupling proteins. J. Mol. Evol.

[b13] Gao CL, Zhu JG, Zhao YP, Chen XH, Ji CB, Zhang CM, Zhu C, Xia ZK, Peng YZ, Guo XR (2010). Mitochondrial dysfunction is induced by the overexpression of UCP4 in 3T3-L1 adipocytes. Int. J. Mol. Med.

[b14] Gao CL, Ni YH, Liu G, Chen XH, Ji CB, Qin DN, Kou CZ, Zhu C, Zhang CM, Xia ZK (2011). UCP4 overexpression improves fatty acid oxidation and insulin sensitivity in L6 myocytes. J. Bioenerg. Biomembr.

[b15] Gonidi M, Athanassiadou AM, Patsouris E, Tsipis A, Dimopoulos S, Kyriakidou V, Chelidonis G, Athanassiadou P (2011). Mitochondrial UCP4 and bcl-2 expression in imprints of breast carcinomas: relationship with DNA ploidy and classical prognostic factors. Pathol. Res. Pract.

[b16] Guevara R, Santandreu FM, Valle A, Gianotti M, Oliver J, Roca P (2009). Sex-dependent differences in aged rat brain mitochondrial function and oxidative stress. Free Radic. Biol. Med.

[b17] Guevara R, Gianotti M, Oliver J, Roca P (2011). Age and sex-related changes in rat brain mitochondrial oxidative status. Exp. Gerontol.

[b104] Guzman JN, Sanchez-Padilla J, Chan CS, Surmeier DJ (2009). Robust pacemaking in substantia nigra dopaminergic neurons. J. Neurosci.

[b19] Guzman JN, Sanchez-Padilla J, Wokosin D, Kondapalli J, Ilijic E, Schumacker PT, Surmeier DJ (2010). Oxidant stress evoked by pacemaking in dopaminergic neurons is attenuated by DJ-1. Nature.

[b20] Hanak P, Jezek P (2001). Mitochondrial uncoupling proteins and phylogenesis–UCP4 as the ancestral uncoupling protein. FEBS Lett.

[b21] Hansford RG, Zorov D (1998). Role of mitochondrial calcium transport in the control of substrate oxidation. Mol. Cell. Biochem.

[b22] Hansford RG, Hogue BA, Mildaziene V (1997). Dependence of H_2_O_2_ formation by rat heart mitochondria on substrate availability and donor age. J. Bioenerg. Biomembr.

[b23] Ho JW, Ho PW, Zhang WY, Liu HF, Kwok KH, Yiu DC, Chan KH, Kung MH, Ramsden DB, Ho SL (2010). Transcriptional regulation of UCP4 by NF-kappaB and its role in mediating protection against MPP+ toxicity. Free Radic. Biol. Med.

[b24] Ho PW, Chan DY, Kwok KH, Chu AC, Ho JW, Kung MH, Ramsden DB, Ho SL (2005). Methyl-4-phenylpyridinium ion modulates expression of mitochondrial uncoupling proteins 2, 4, and 5 in catecholaminergic (SK-N-SH) cells. J. Neurosci. Res.

[b25] Ho PW, Chu AC, Kwok KH, Kung MH, Ramsden DB, Ho SL (2006). Knockdown of uncoupling protein-5 in neuronal SH-SY5Y cells: effects on MPP+-induced mitochondrial membrane depolarization, ATP deficiency, and oxidative cytotoxicity. J. Neurosci. Res.

[b26] Ho PW-L, W.-M. Ho J, Tse H-M, H.-F. So D, C.-W. Yiu D, Liu H-F, Chan K-H, H.-W. Kung M, Ramsden DB, Ho S-L (2012). Uncoupling protein-4 (UCP4) increases ATP supply by interacting with mitochondrial Complex II in neuroblastoma cells. PLoS One.

[b27] Horvath TL, Warden CH, Hajos M, Lombardi A, Goglia F, Diano S (1999). Brain uncoupling protein 2: uncoupled neuronal mitochondria predict thermal synapses in homeostatic centers. J. Neurosci.

[b28] Huang PS, Son JH, Abbott LC, Winzer-Serhan UH (2011). Regulated expression of neuronal SIRT1 and related genes by aging and neuronal beta2-containing nicotinic cholinergic receptors. Neuroscience.

[b29] Huang SG, Klingenberg M (1995). Fluorescent nucleotide derivatives as specific probes for the uncoupling protein: thermodynamics and kinetics of binding and the control by pH. Biochemistry.

[b30] Ivanova MV, Hoang T, Mcsorley FR, Krnac G, Smith MD, Jelokhani-Niaraki M (2010). A comparative study on conformation and ligand binding of the neuronal uncoupling proteins. Biochemistry.

[b31] Jastroch M, Divakaruni AS, Mookerjee S, Treberg JR, Brand MD (2010). Mitochondrial proton and electron leaks. Essays Biochem.

[b32] Jezek P (2002). Possible physiological roles of mitochondrial uncoupling proteins–UCPn. Int. J. Biochem. Cell. Biol.

[b33] Jezek P, Urbankova E (2000). Specific sequence of motifs of mitochondrial uncoupling proteins. IUBMB Life.

[b34] Kim-Han JS, Dugan LL (2005). Mitochondrial uncoupling proteins in the central nervous system. Antioxid. Redox Signal.

[b35] Kim-Han JS, Reichert SA, Quick KL, Dugan LL (2001). BMCP1: a mitochondrial uncoupling protein in neurons which regulates mitochondrial function and oxidant production. J. Neurochem.

[b36] Kitahara T, Li HS, Balaban CD (2004). Localization of the mitochondrial uncoupling protein family in the rat inner ear. Hear. Res.

[b37] Klingenberg M (1988). Nucleotide binding to uncoupling protein. Mechanism of control by protonation. Biochemistry.

[b38] Kondou S, Hidaka S, Yoshimatsu H, Tsuruta Y, Itateyama E, Sakata T (2000). Molecular cloning of rat brain mitochondrial carrier protein-1 cDNA and its up-regulation during postnatal development. Biochim. Biophys. Acta.

[b39] Korshunov SS, Skulachev VP, Starkov AA (1997). High protonic potential actuates a mechanism of production of reactive oxygen species in mitochondria. FEBS Lett.

[b40] Kwok KH, Ho PW, Chu AC, Ho JW, Liu HF, Yiu DC, Chan KH, Kung MH, Ramsden DB, Ho SL (2010). Mitochondrial UCP5 is neuroprotective by preserving mitochondrial membrane potential, ATP levels, and reducing oxidative stress in MPP+ and dopamine toxicity. Free Radic. Biol. Med.

[b41] Lanzillotta A, Sarnico I, Ingrassia R, Boroni F, Branca C, Benarese M, Faraco G, Blasi F, Chiarugi A, Spano et al P (2010). The acetylation of RelA in Lys310 dictates the NF-kappaB-dependent response in post-ischemic injury. Cell Death Dis.

[b42] Lengacher S, Magistretti PJ, Pellerin L (2004). Quantitative rt-PCR analysis of uncoupling protein isoforms in mouse brain cortex: methodological optimization and comparison of expression with brown adipose tissue and skeletal muscle. J. Cereb. Blood Flow Metab.

[b43] Liu D, Chan SL, De Souza-Pinto NC, Slevin JR, Wersto RP, Zhan M, Mustafa K, De Cabo R, Mattson MP (2006). Mitochondrial UCP4 mediates an adaptive shift in energy metabolism and increases the resistance of neurons to metabolic and oxidative stress. Neuromolecular Med.

[b44] Mao W, Yu XX, Zhong A, Li W, Brush J, Sherwood SW, Adams SH, Pan G (1999). UCP4, a novel brain-specific mitochondrial protein that reduces membrane potential in mammalian cells. FEBS Lett.

[b45] Mathis JM, Simmons DM, He X, Swanson LW, Rosenfeld MG (1992). Brain 4: a novel mammalian POU domain transcription factor exhibiting restricted brain-specific expression. EMBO J.

[b46] Mitchell P (1961). Coupling of phosphorylation to electron and hydrogen transfer by a chemi-osmotic type of mechanism. Nature.

[b47] Mitchell P (1966). Chemiosmotic coupling in oxidative and photosynthetic phosphorylation. Biol. Rev. Camb. Philos. Soc.

[b48] Mouaffak F, Kebir O, Bellon A, Gourevitch R, Tordjman S, Viala A, Millet B, Jaafari N, Olie JP, Krebs MO (2011). Association of an UCP4 (SLC25A27) haplotype with ultra-resistant schizophrenia. Pharmacogenomics.

[b49] Nakase T, Yoshida Y, Nagata K (2007). Amplified expression of uncoupling proteins in human brain ischemic lesions. Neuropathology.

[b50] Naudi A, Caro P, Jove M, Gomez J, Boada J, Ayala V, Portero-Otin M, Barja G, Pamplona R (2007). Methionine restriction decreases endogenous oxidative molecular damage and increases mitochondrial biogenesis and uncoupling protein 4 in rat brain. Rejuvenation Res.

[b51] O’Donovan KJ, Tourtellotte WG, Millbrandt J, Baraban JM (1999). The EGR family of transcription-regulatory factors: progress at the interface of molecular and systems neuroscience. Trends Neurosci.

[b52] Palmieri F (1994). Mitochondrial carrier proteins. FEBS Lett.

[b53] Palmieri F (2004). The mitochondrial transporter family (SLC25): physiological and pathological implications. Pflugers Arch.

[b54] Pfeiffer M, Kayzer EB, Yang X, Abramson E, Kenaston MA, Lago CU, Lo HH, Sedensky MM, Lunceford A, Clarke CF (2011). *Caenorhabditis elegans* UCP4 protein controls Complex II-mediated oxidative phosphorylation through succinate transport. J. Biol. Chem.

[b55] Pizzi M, Spano P (2006). Distinct roles of diverse nuclear factor-[kappa]B complexes in neuropathological mechanisms. Eur. J. Pharmacol.

[b56] Pizzi M, Goffi F, Boroni F, Benarese M, Perkins SE, Liou HC, Spano P (2002). Opposing roles for NF-kappa B/Rel factors p65 and c-Rel in the modulation of neuron survival elicited by glutamate and interleukin-1beta. J. Biol. Chem.

[b57] Pizzi M, Sarnico I, Boroni F, Benarese M, Steimberg N, Mazzoleni G, Dietz GP, Bahr M, Liou HC, Spano PF (2005). NF-kappaB factor c-Rel mediates neuroprotection elicited by mGlu5 receptor agonists against amyloid beta-peptide toxicity. Cell Death Differ.

[b58] Quentien MH, Vieira V, Menasche M, Dufier JL, Herman JP, Enjalbert A, Abitbol M, Brue T (2011). Truncation of PITX2 differentially affects its activity on physiological targets. J. Mol. Endocrinol.

[b59] Sakamoto K, Karelina K, Obrietan K (2011). CREB: a multifaceted regulator of neuronal plasticity and protection. J. Neurochem.

[b60] Sanchez-Blanco A, Fridell YW, Helfand SL (2006). Involvement of Drosophila uncoupling protein 5 in metabolism and aging. Genetics.

[b61] Sanchis D, Fleury C, Chomiki N, Goubern M, Huang Q, Neverova M, Gregoire F, Easlick J, Raimbault S, Levi-Meyrueis C, et al (1998). BMCP1, a novel mitochondrial carrier with high expression in the central nervous system of humans and rodents, and respiration uncoupling activity in recombinant yeast. J. Biol. Chem.

[b62] Santandreu FM, Valle A, Fernandez De Mattos S, Roca P, Oliver J (2009). Hydrogen peroxide regulates the mitochondrial content of uncoupling protein 5 in colon cancer cells. Cell Physiol. Biochem.

[b63] Sastre-Serra J, Valle A, Company MM, Garau I, Oliver J, Roca P (2010). Estrogen down-regulates uncoupling proteins and increases oxidative stress in breast cancer. Free Radic. Biol. Med.

[b64] Senapedis WT, Kennedy CJ, Boyle PM, Silver PA (2011). Whole genome siRNA cell-based screen links mitochondria to Akt signaling network through uncoupling of electron transport chain. Mol. Biol. Cell.

[b65] Skulachev VP (1996). Role of uncoupled and non-coupled oxidations in maintenance of safely low levels of oxygen and its one-electron reductants. Q. Rev. Biophys.

[b66] Skulachev VP (1997). Membrane-linked systems preventing superoxide formation. Biosci. Rep.

[b67] Skulachev VP (1998). Uncoupling: new approaches to an old problem of bioenergetics. Biochim. Biophys. Acta.

[b68] Smorodchenko A, Rupprecht A, Sarilova I, Ninnemann O, Brauer AU, Franke K, Schumacher S, Techritz S, Nitsch R, Schuelke M (2009). Comparative analysis of uncoupling protein 4 distribution in various tissues under physiological conditions and during development. Biochim. Biophys. Acta.

[b69] Smorodchenko A, Rupprecht A, Fuchs J, Gross J, Pohl EE (2011). Role of mitochondrial uncoupling protein 4 in rat inner ear. Mol. Cell. Neurosci.

[b70] Sokolova IM, Sokolov EP (2005). Evolution of mitochondrial uncoupling proteins: novel invertebrate UCP homologues suggest early evolutionary divergence of the UCP family. FEBS Lett.

[b71] Stout AK, Raphael HM, Kanterewicz BI, Klann E, Reynolds IJ (1998). Glutamate-induced neuron death requires mitochondrial calcium uptake. Nat. Neurosci.

[b72] Sullivan PG, Rippy NA, Dorenbos K, Concepcion RC, Agarwal AK, Rho JM (2004). The ketogenic diet increases mitochondrial uncoupling protein levels and activity. Ann. Neurol.

[b86] Surmeier DJ, Guzman JN, Sanchez-Padilla J, Schumacker PT (2011). The role of calcium and mitochondrial oxidative stress in the loss of substantia nigra pars compacta dopamineric neurons in Parkinson's disease. Neuroscience.

[b73] Szolnoki Z, Kondacs A, Mandi Y, Bodor A, Somogyvari F (2009). A homozygous genetic variant of mitochondrial uncoupling protein 4 exerts protection against the occurrence of multiple sclerosis. Neuromol. Med.

[b74] Szolnoki Z, Kondacs A, Mandi Y, Bodor A, Somogyvari F (2011). A homozygous genetic variant of mitochondrial uncoupling protein 4 affects the occurrence of leukoaraiosis. Acta Neurol. Scand.

[b75] Votyakova TV, Reynolds IJ (2001). DeltaPsi(m)-Dependent and -independent production of reactive oxygen species by rat brain mitochondria. J. Neurochem.

[b76] Wei Z, Chigurupati S, Bagsiyao P, Henriquez A, Chan SL (2009). The brain uncoupling protein UCP4 attenuates mitochondrial toxin-induced cell death: role of extracellular signal-regulated kinases in bioenergetics adaptation and cell survival. Neurotox. Res.

[b77] Wu Z, Zhang J, Zhao B (2009). Superoxide anion regulates the mitochondrial free Ca2+ through uncoupling proteins. Antioxid. Redox Signal.

[b78] Xia C, Liu JZ, Xu Y (2008). [Effects of GDP on the activity and expression of mitochondrial uncoupling proteins in rat brain in vitro.]. Sheng Li Xue Bao.

[b79] Yang X, Pratley RE, Tokraks S, Tataranni PA, Permana PA (2002). UCP5/BMCP1 transcript isoforms in human skeletal muscle: relationship of the short-insert isoform with lipid oxidation and resting metabolic rates. Mol. Genet. Metab.

[b80] Yasuno K, Ando S, Misumi S, Makino S, Kulski JK, Muratake T, Kaneko N, Amagane H, Someya T, Inoko H, et al (2007). Synergistic association of mitochondrial uncoupling protein (UCP) genes with schizophrenia. Am. J. Med. Genet. B.

[b81] Yoneda Y, Kuramoto N, Kitayama T, Hinoi E (2001). Consolidation of transient ionotropic glutamate signals through nuclear transcription factors in the brain. Prog. Neurobiol.

[b82] Yonezawa T, Haga S, Kobayashi Y, Katoh K, Obara Y (2009). Saturated fatty acids stimulate and insulin suppresses BMCP1 expression in bovine mammary epithelial cells. Biochem. Biophys. Res. Commun.

[b83] Yu XX, Barger JL, Boyer BB, Brand MD, Pan G, Adams SH (2000a). Impact of endotoxin on UCP homolog mRNA abundance, thermoregulation, and mitochondrial proton leak kinetics. Am. J. Physiol. Endocrinol. Metab.

[b84] Yu XX, Mao W, Zhong A, Schow P, Brush J, Sherwood SW, Adams SH, Pan G (2000b). Characterization of novel UCP5/BMCP1 isoforms and differential regulation of UCP4 and UCP5 expression through dietary or temperature manipulation. FASEB J.

[b85] Zhang M, Wang B, Ni YH, Liu F, Fei L, Pan XQ, Guo M, Chen RH, Guo XR (2006). Overexpression of uncoupling protein 4 promotes proliferation and inhibits apoptosis and differentiation of preadipocytes. Life Sci.

